# Supplementary material for: Spatio-Temporal Patterns of the International Merger and Acquisition Network
Source: Sci Rep. 2017 Sep 7;7:10789. doi: 10.1038/s41598-017-10779-z (PMC5589942; doi:10.1038/s41598-017-10779-z)
Supplement: Supplementary file 1 — Supplementary Information [file 41598_2017_10779_MOESM1_ESM.pdf]

Spatio-Temporal Patterns of the International Merger and Acquisition Network

Supplementary Information

Marco Dueñas[1,*], Rossana Mastrandrea[2], Matteo Barigozzi[3], and Giorgio Fagiolo[4]

[1]Department of Economics, International Trade and Social Policy, Universidad de Bogotá Jorge Tadeo Lozano, Bogotá
[2]IMT School for Advanced Studies, Lucca
[3]Department of Statistics, London School of Economics and Political Science, London
[4]Istituto di Economia, Scuola Superiore Sant'Anna, Pisa
[*]E-mail: marcoa.duenase@utadeo.edu.co (corresponding author)

Country Groups

Group 1

Argentina; Australia; Belgium; Bermuda; Brazil; British Virgin; Canada; Chile; China; Denmark; Finland; France; Germany; Hong Kong; India; Ireland-Rep; Israel; Italy; Japan; Luxembourg; Malaysia; Mexico; Netherlands; New Zealand; Norway; Philippines; Portugal; Singapore; South Africa; South Korea; Spain; Sweden; Switzerland; Taiwan; Thailand; United Kingdom; United States.

Group 2

Antigua and Barbuda; Armenia; Austria; Azerbaijan; Bahamas; Bahrain; Bangladesh; Barbados; Belize; Bolivia; Bosnia and Herzegovina; Botswana; Brunei; Bulgaria; Cambodia; Cayman Islands; Colombia; Congo; Costa Rica; Croatia; Cuba; Cyprus; Czech Republic; Dominican Rep; Ecuador; Egypt; El Salvador; Estonia; Fiji; Ghana; Gibraltar; Greece; Guam; Guatemala; Guernsey; Hungary; Iceland; Indonesia; Iraq; Isle of Man; Ivory Coast; Jamaica; Jersey; Jordan; Kazakhstan; Kenya; Kuwait; Latvia; Lebanon; Libya; Liechtenstein; Lithuania; Macao; Macedonia; Malta; Mauritius; Monaco; Mongolia; Montenegro; Morocco; New Caledonia; Nigeria; Oman; Pakistan; Panama; Papua New Guinea; Peru; Poland; Puerto Rico; Qatar; Romania; Russian Federation; Saudi Arabia; Serbia; Seychelles; Slovak Rep; Slovenia; Sri Lanka; Sudan; Trinidad & Tobago; Tunisia; Turkey; Turks/Caicos; Ukraine; United Arab Emirates; Uruguay; US Virgin Is; Uzbekistan; Venezuela; Vietnam; Western Samoa; Zambia; Zimbabwe.

Group 3

Afghanistan; Albania; Algeria; American Samoa; Andorra; Angola; Anguilla; Aruba; Belarus; Benin; Bhutan; Burkina Faso; Burundi; Cameroon; Cape Verde; Central African Republic; Chad; Comoros; Cook Islands; Democratic Republic of Congo; Djibouti; Dominica; Equatorial Guinea; Eritrea; Ethiopia; Falkland Islands; Faroe Islands; French Polynesia; Gabon; Gambia; Georgia; Greenland; Grenada; Guadeloupe; Guinea; Guinea-Bissau; Guyana; Haiti; Honduras; Iran; Kiribati; Kyrgyzstan; Laos; Lesotho; Liberia; Madagascar; Malawi; Maldives; Mali; Marshall Islands; Martinique; Mauritania; Mayotte; Micronesia; Moldova; Mozambique; Myanmar(Burma); N. Mariana; Namibia'; Nauru; Nepal; Nicaragua; Niger; Niue; Norfolk Island; North Korea; Palau; Palestine; Paraguay; Reunion; Rwanda; Saint Vincent and the Grenadines; San Marino; Sao Tome; Senegal; Sierra Leone; Solomon Islands; Somalia; St Kitts & Nevis; St Lucia; Suriname; Swaziland; Syria; Tajikistan; Tanzania; Timor-Leste; Togo; Tokelau; Tonga; Turkmenistan; Tuvalu; Uganda; Vanuatu; Yemen.

Network Topology

We test whether the empirical probability distribution of node out-degree differ from that of node in-degree (we call this set of tests "between" tests). Furthermore, we test whether in-degree probability distributions differ across the years (we call this set of tests "within" tests), and similarly for out-degree probability distributions. Table 1 shows two-sample Kolmogorov-Smirnov (K-S) tests within and between the distributions of ND_{in} and ND_{out} in different years. In the case of the distribution of ND_{in}, the null hypothesis of K-S test is almost always rejected, allowing us to conclude that in-degree profiles are quite unstable. Conversely, in the case of the distribution of ND_{out}, the null hypothesis of the K-S test is most frequently non rejected. This is an expected result because the IMAN is very target oriented and suggests that out-degree profiles are quite similar in different

periods. Finally, the null hypothesis of the K-S test to compare the in-degree and out-degree distributions is always rejected. This highlights that out- and in-degree profiles are very different.

Table 1. Two-sample Kolmogorov-Smirnov Tests within and between in- and out-degree distributions in different years.

	Within test of In-degree				Within test of Out-degree				Between test of In-degree vs. Out-degree				
Column	(1)	(2)	(3)	(4)	(5)	(6)	(7)	(8)	(9)	(10)	(11)	(12)	(13)
Year	2000	2003	2007	2010	2000	2003	2007	2010	1995	2000	2003	2007	2010
1995	0.12	0.05***	0.12	0.11	0.07*	0.06**	0.13	0.10	0.31	0.24	0.25	0.30	0.27
	(0.35)	(1.00)	(0.30)	(0.42)	(0.92)	(0.98)	(0.25)	(0.60)	(0.00)	(0.00)	(0.00)	(0.00)	(0.00)
2000		0.10	0.08	0.07		0.05***	0.07*	0.05***		0.23	0.24	0.29	0.26
		(0.53)	(0.70)	(0.89)		(1.00)	(0.91)	(0.99)		(0.00)	(0.00)	(0.00)	(0.00)
2003			0.09	0.09			0.08	0.06*			0.21	0.26	0.23
			(0.62)	(0.64)			(0.79)	(0.92)			(0.00)	(0.00)	(0.00)
2007				0.07				0.04***				0.32	0.30
				(0.85)				(1.00)				(0.00)	(0.00)
2010													0.32
													(0.00)

Notes: The null hypotheses tested in columns (1-4) and (5-8) are that the observed distributions come from the same distributions, within in-degree and out-degree distributions, respectively. The null hypotheses tested in columns (9-13) are that the observed distributions between in-degree and out-degree come from the same distributions. The p-values are reported in parenthesis. Significance level: *** p<0.01, ** p<0.05, * p<0.10.

Table 2 presents the top-10 ranking of countries with high node degree (*ND*) and node strength (*NS*). Here, we are interested in the co-occurrence of rankings of total country's M&As (i.e, node-strength) and the country's number of partners (i.e., node degree), distinguishing among in- and out-oriented statistics. Comparing within statistics across different years, for both *ND* and *NS*, we observe high co-occurrence of several high-income economies. Although the order in which they appear changes from year to year, some countries, such as the USA and GBR, appear recurrently in the top of the rankings. Interestingly, we observe in the rankings a few emergent economies, as China, which appears in 2000 as the target of high-volume inflows and in 2010 as an acquirer with very high outflows.

Table 2. Node degree and node strength rankings.

1995		2000		2003		2007		2010	
Node Degree									
in	out	in	out	in	out	in	out	in	out
USA	USA	USA	USA	USA	USA	USA	GBR	GBR	USA
GBR	GBR	GBR	GBR	GBR	GBR	GBR	USA	USA	GBR
DEU	CAN	CAN	FRA	DEU	CAN	CAN	AUS	AUS	CAN
FRA	DEU	BRA	DEU	AUS	NLD	DEU	CAN	CAN	AUS
AUS	NLD	AUS	NLD	FRA	DEU	AUS	NLD	FRA	NLD
HKG	FRA	FRA	ITA	CHI	FRA	CHI	FRA	DEU	DEU
CAN	JPN	HKG	CAN	IND	CHE	IND	ESP	NLD	SGP
ITA	SGP	NLD	SWE	NLD	ESP	NLD	RUS	ITA	SWE
NLD	AUS	DEU	BEL	HKG	JPN	RUS	DEU	CHI	HKG
ESP	CHE	JPN	JPN	CHE	HKG	FRA	JPN	IND	IND
Node-Strength									
in	out	in	out	in	out	in	out	in	out
USA	USA	USA	GBR	USA	USA	USA	USA	USA	USA
GBR	GBR	DEU	FRA	GBR	GBR	GBR	GBR	GBR	GBR
AUS	DEU	GBR	USA	DEU	DEU	CAN	FRA	BRA	CAN
CAN	CAN	CAN	DEU	FRA	CAN	DEU	DEU	DEU	CHI
SWE	FRA	CHI	ESP	CHE	AUS	NLD	CAN	CAN	JPN
FRA	CHE	FRA	HKG	ITA	NLD	FRA	ITA	AUS	IND
DEU	NLD	NLD	CHE	JPN	FRA	ESP	NLD	FRA	NLD
ITA	SWE	BRA	NLD	NLD	CHE	AUS	ESP	NLD	DEU
CHE	ITA	ESP	CAN	AUS	JPN	ITA	AUS	CHI	FRA
NLD	BEL	SWE	SWE	ESP	MEX	CHE	SWE	ESP	ESP

The rankings suggest that there exists a positive relation between the number of targets and the outflow volumes, and, similarly, between the number of acquiring countries and the inflow volumes. Figure 1a and Figure 1b show the scatter plots between node degree and node strength.

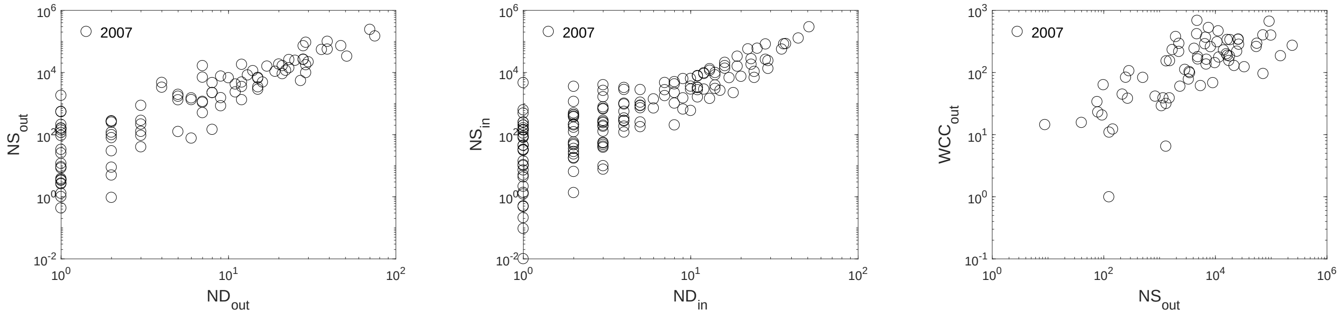

(a) **(b)** **(c)**

Figure 1. **Scatter plots of network statistics in 2017.**

Table 3. Binary and weighted clustering coefficient rankings.

1995	2000	2003	2007	2010
		BCC_{out}		
Cuba	Puerto Rico	Costa Rica	Dominican Rep.	Puerto Rico
Barbados	Dominican Rep.	Guernsey	Costa Rica	Barbados
British Virgin	Guatemala	Luxembourg	Guyana	Costa Rica
Ireland Rep.	Bolivia	Greece	Bolivia	Jersey
Luxembourg	Isle of Man	Estonia	Isle of Man	Isle of Man
		BCC_{in}		
Kuwait	Isle of Man	Bahamas	Liechtenstein	Macao
Indonesia	Iceland	Guernsey	Oman	Philippines
Luxembourg	Bahrain	Jersey	Vietnam	Indonesia
Denmark	Papua New Guinea	Netherlands Antilles	Western Samoa	Qatar
Israel	China	Monaco	Jersey	New Zealand
		BCC_{cyc}		
Cuba	Puerto Rico	Trinidad & Tobago	Bahamas	US Virgin Is.
Uruguay	Antigua and Barbuda	Costa Rica	Barbados	Uruguay
British Virgin	Panama	Peru	Costa Rica	Liechtenstein
Netherlands Antilles	Venezuela	Guernsey	Gibraltar	Kazakhstan
Luxembourg	Guernsey	Monaco	Botswana	Macao
		BCC_{mid}		
Bahamas	Puerto Rico	Trinidad & Tobago	Bahamas	Liechtenstein
Netherlands Antilles	Antigua and Barbuda	Costa Rica	El Salvador	Seychelles
Ghana	Panama	Uruguay	Costa Rica	Kazakhstan
Luxembourg	Guernsey	Guernsey	Montenegro	Macao
Denmark	Isle of Man	Jersey	Botswana	Bangladesh
		WCC_{out}		
Czechoslovakia**	Germany	Luxembourg	Belarus	Papua New Guinea
Ireland Rep.	Luxembourg	Italy	Qatar	Puerto Rico
Sweden	Spain	Greece	El Salvador	Mexico
Australia	Belgium	Israel	Puerto Rico	Brazil
Brazil	Netherlands	Portugal	Hungary	Bermuda
		WCC_{in}		
South Africa	Papua New Guinea	Ireland-Rep	Argentina	Qatar
Italy	France	Israel	Germany	New Zealand
Saudi Arabia	Iceland	Netherlands Antilles	Iceland	Bermuda
France	Bermuda	Monaco	Norway	Ireland Rep.
Sweden	Switzerland	Guernsey	New Zealand	Israel
		WCC_{cyc}		
Netherlands Antilles	Germany	Trinidad & Tobago	Bahamas	Bermuda
Argentina	France	Israel	Iceland	Kazakhstan
Sweden	Luxembourg	Monaco	Germany	Brazil
Switzerland	Switzerland	Luxembourg	Italy	Ireland Rep.
Italy	Bermuda	Italy	France	Mexico
		WCC_{mid}		
Netherlands Antilles	Germany	Trinidad & Tobago	Iceland	Kazakhstan
Sweden	France	Luxembourg	Bahamas	Bermuda
Switzerland	Guernsey	Israel	Germany	Mexico
France	Spain	Italy	El Salvador	Brazil
Italy	Luxembourg	Ireland Rep.	France	United States

** Although Czechoslovakia split into two sovereign states in 1993, still in 1995 several firms appear in the database as based in Czechoslovakia.

In contrast, we observe that clustering patterns are more heterogeneous. There are neither well established nor persistent hubs in the network. Table 3 shows the top-5 positions for all directed clustering types. The heterogeneity along time and between clustering types is the reason behind the weak and fluctuating correlations among clustering and node degree and node strengths. Figure 1c shows the scatter plot between node strength and clustering correlation.

Spatial Analysis

The left panel of Figure 2a shows the kernel-density estimation of the distribution of the entries of the geographical-distance matrix (in logs) in 2007. The estimated distribution is left-skewed and bimodal with a higher peak at farther distances. These patterns are robust over time. The Q-Q plots in Figure 2b compare the distributions for different years. We observe a linear relation among the quantiles of the distributions, which suggests that the shapes of the distributions are quite stable.

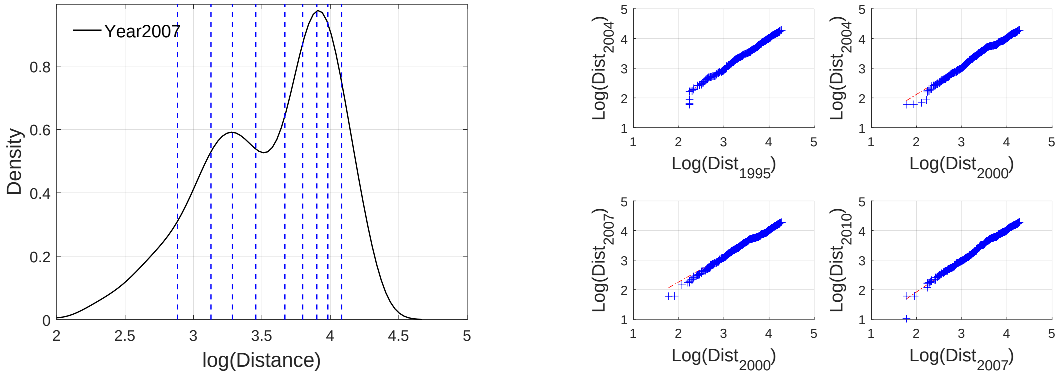

(a) **(b)**

Figure 2. The distribution of geographical distance between country pairs with M&As. (a) Kernel estimation for 2007, vertical lines indicate the deciles of the distribution. **(b)** Q-Q plots comparing distributions of different years.
